# Defective Awareness of Person-Recognition Disorders Through Face, Voice and Name in Right and Left Variants of Semantic Dementia: A Pilot Study

**DOI:** 10.3390/brainsci15050504

**Published:** 2025-05-15

**Authors:** Simona Luzzi, Oscar Prata, Guido Gainotti

**Affiliations:** 1 Cognitive and Behavioural Neurology Unit, Department of Experimental and Clinical Medicine, Polytechnic University of Marche, 60126 Ancona, Italy; o.prata@pm.univpm.it; 2 Centre for Neuropsychological Research, Department of Neurosciences, Catholic University of Rome, 00168 Rome, Italy; guido.gainotti@unicatt.it

**Keywords:** semantic dementia, awareness, anosognosia, person-recognition disorders

## Abstract

**Background/Objectives**: The aim of this investigation consisted of evaluating if the prevalence of anosognosia in right-brain-damaged patients is greater for tasks in which the right hemisphere plays a dominant role and if this prevalence is at least in part due to automatic processing mechanisms typical of this hemisphere. **Methods**: We assessed defective awareness of person-recognition disorders in 14 patients with the right variant (rv-SD) and 15 with the left variant (lv-SD) of Semantic Dementia. A battery exploring person-recognition disorders through familiarity judgement of faces, voices and names was applied. In patients with pathological performance in one of these modalities, anosognosia was assessed comparing the patients’ subjective judgment to the objective result of their performance (objective evaluation) and to the subjective judgment given by an informed caregiver (external comparison). **Results**: In the comparison between subjective awareness and objective scores in the various person-recognition modalities, only anosognosia for face recognition disorders was significantly more frequent of in patients with rv-SD. When compared to their caregivers, subjects with rv-SD were significantly less aware than caregivers of their difficulties only on face recognition. On the contrary, patients with a lv-SD showed a greater (non-significant) trend to be unaware of their name recognition deficit. **Conclusions**: These data show that the prevalence of anosognosia in right-brain-damaged patients is greater for face recognition in which the right hemisphere plays a dominant role and that this prevalence is at least in part due to automatic processing mechanisms (evocation of familiarity feelings) typical of this hemisphere.

## 1. Introduction

The aim of the present investigation consisted of trying to clarify the links between right-hemispheric lesions and a new form of anosognosia (namely unawareness of person-recognition disorders through face, voice and name). Clinical and theoretical reasons motivated our study. The clinical reason was to verify whether the hypothesis of a prevalent involvement of the right hemisphere in patients with anosognosia was applicable to per-son recognition disorders that had never been explored before. This privileged relationship between anosognosia and right-hemisphere lesions was proposed by Babinski [1] with his pioneering studies on the unawareness of left-sided hemiplegia but was later subjected to some criticism. It could, therefore, be interesting to evaluate if right-hemisphere lesions contribute equally to anosognosia in all forms of people-recognition disorders. In fact, most authors (e.g., [2,3,4,5,6,7,8,9,10]) maintain that disorders of person recognition through face and voice are mainly underpinned by lesions of the right hemisphere, whereas defects of person recognition through personal name are mainly due to left-hemisphere damage. This dissociation, that is frequently observed in patients with the right variant (rvSD) and left variant (lvSD) of Semantic Dementia (SD), (e.g., [2,3,5,7,10]) could be considered as a sort of ‘experimentum nature’, allowing one to test some complex cognitive models on the relationships between anosognosia and right-hemisphere damage. 

In the first part of this introduction, we will therefore summarize some relevant aspects of the relationships between anosognosia and right-hemisphere lesions, whereas in the second part we will dwell on theoretical questions that could be clarified by exploring anosognosia for person-recognition disorders through face, voice and name in patients with the rvSD or lvSD.

**1.**  **Links between anosognosia and right-hemisphere lesions**

In his clinical studies on the unawareness of left-sided hemiplegia, Babinski [1] had speculated that anosognosia may be specific to right-hemisphere lesions [11]. This notion was systematically confirmed by several anatomo-clinical investigations (see review in Galin [12]).

In more recent times, however, this notion has been submitted to a more critical scrutiny by studies that have supported or refuted the assumption of a privileged relation between anosognosia and right-hemispheric lesions. Therefore, some authors (e.g., [13]) have suggested that the prevalence of anosognosia for left hemiplegia could be due to a sampling bias, i.e., the exclusion from the study of patients with extensive lesions on the left side, because the presence of severe language disorders had made these patients un-testable. This suggestion was, however, inconsistent with results of investigations (e.g., [14,15]) that had used injection of barbiturate (Wada test) to induce hemiplegia under more controlled conditions, where it was found that very few participants showed unawareness for motor disorders after suppression of left-hemisphere activity. 

The notion of a strong link between anosognosia and right-hemisphere lesions was also reinforced by authors (e.g., [16,17,18,19,20]) who noted that the incidence and severity of anosognosia, such as unilateral spatial neglect, were particularly high for disorders that are typically due to right-hemisphere lesions. 

On the other hand, the hypothesis of a strong relation between unawareness and right-hemisphere lesions has been undermined by the observation that anosognosia is a partly selective defect, which may concern only some disabilities, sparing other disorders presented by the same patient (e.g., [21,22]). This dissociation suggests, that in anosognosia, brain damage could selectively impact the self-monitoring process of specific physical or cognitive functions rather than affecting a general control from the frontal lobes, or a unitary mechanism subsumed by the right hemisphere [8,23,24].

Furthermore, other investigations suggested that anosognosia could be a heterogeneous phenomenon, influenced by sensory, motor, cognitive and emotional factors. For instance, Levine [25] maintained that interruption of a sensory pathway at any level may not be associated with any immediate sensory experience that uniquely specifies the defect. He assumed that the sensory loss must be discovered by a process of self-observation and inference that can be precluded by severe cognitive impairment. Other authors (e.g., [16,17,18]) showed that the prevalence of anosognosia for hemiplegia in right brain-damaged patients can be partly explained by the concomitant presence of unilateral neglect and somatosensory disorders. Still other authors (e.g., [18,26,27,28]) stressed the importance of emotional (denial) factors, drawing on the Babinski’s [29] paradoxical observation that some patients who ignored their severe hemiplegia had been afraid for many years of this pathological condition. This dramatic change from anxious expectations to lack of concern suggested that motivational factors might force some patients to deny a condition that they are unable to accept. 

On the basis of these observations, many authors acknowledge that the presence of a right-hemispheric lesion is not a necessary condition for the development of anosognosia and that defective awareness is also determined by the disruption of selective self-monitoring processes, which is intrinsic to the organization of the disrupted functions. Much more difficult, however, remains the task of evaluating the role of these selective self-monitoring processes and of the nonverbal functioning of the right hemisphere in generating a reduced self-awareness.

**2.**  **Insights from this study on the relationship between anosognosia and right-hemisphere lesions**

A comparative study of the relationships between right-hemispheric lesions and anosognosia of forms of person-recognition disorders through face and voice (mainly sub-tended by the right hemisphere) and through name (left hemisphere) could perhaps help to clarify two questions: (a) the link between anosognosia and right-hemisphere lesions and (b) the right-hemisphere mechanisms that contribute to the development of anosognosia.

Comparative studies of anosognosia for structurally similar tasks, based respectively on verbal and non-verbal material, subtended by the left and right hemisphere, could in fact help one to understand if there is a link between the processing of a specific material and the awareness of this processing. In this case, we should observe a prevalent anosognosia of face and voice recognition disorders in rvSD and of name recognition disorders in lvSD. On the other hand, theoretical models of hemispheric asymmetries could allow one to advance suggestions on the mechanisms of the right hemisphere that could contribute to its prevalent role in the development of anosognosia. In a recent reappraisal of the problem of brain asymmetries, Gainotti [30,31] has indeed suggested that two functional aspects of the right hemisphere, linked to its non-verbal organization (namely its automatic processing modes and its partly unconscious functioning), could explain this lack of awareness. The existence of automatic processing modalities in patients with right-hemisphere lesions are documented by the disruption of automatic mechanisms in two typical right-hemispheric syndromes, such as unilateral spatial neglect and prosopagnosia. Neglect of the left half space is indeed due to the automatic capture of attention by stimuli lying in the right (contralateral) half space, with sparing of intentional orienting of attention towards the same side of space [32,33]. Similarly, face recognition disorders are mainly due to an impaired automatic generation of face familiarity feelings that trigger the search for the corresponding biographic information within the semantic system [4,8,10,34]. This greater involvement of the right hemisphere in the automatic aspects of brain functions could influence its level of consciousness because, according to Shiffrin and Schneider [35], “auto-matic” mechanisms tend to be associated with less conscious processing whereas “inten-tional/controlled” mechanisms are linked to a more conscious functioning. 

From the theoretical point of view, the greater role of the right hemisphere in anosognosia could be due to two aspects of its functional organization (the absence of language and the dominance for emotions) that could impact of its level of conscious awareness. As for the first issue, many authors (e.g., [36,37,38,39]) have underlined that the levels of consciousness and of intentionality are general aspects of thought that can be shaped by language. As for the second issue, it is well known that emotionally laden stimuli can be detected, processed and learned without conscious awareness through a right hemispheric subcortical pathway, mediating unconscious emotional learning (e.g., [40,41,42]). Taken together, these theoretical, clinical and experimental reasons could explain the poorly conscious working mode typical of the right hemisphere and the defective awareness of emotionally laden conditions observed in patients with right brain lesions.

**3.**  **‘Automatic functioning’ and the ‘non-verbal organization’ of the right hemisphere: insight from defective awareness of person-recognition disorders in SD**

To evaluate the impact that the ‘automatic functioning’ and the ‘non-verbal organiza-tion’ of the right hemisphere could have on defective awareness, it could be useful to find clinical conditions in which these two factors are dissociated. This could happen in simi-lar tasks, based on the same automatic functioning effects, but differentially supported by the right and left hemisphere, because this dissociation should help to clarify the burden on anosognosia of automatic functioning and of ‘non-verbal organization’ of the right hemisphere. Starting from these premises, we hypothesized that tasks of person recognition through face, voice and name, that are frequently impaired in patients with the rvSD or lvSD (e.g., [2,3,4,6,10,43]) could fit these criteria. The automatic generation of modality-specific familiarity feelings is indeed requested by each of these tasks, but the familiarity feelings of non-verbal tasks of face and voice recognition are mainly supported by the right anterior temporal lobe (rATL), whereas the familiarity feelings of the verbal task of name recognition is supported by the left ATL (e.g., [4,8,10,44]). 

Since no study of the unawareness of person-recognition disorders through face, voice and name has been conducted up to date in patients with the rvSD or lvSD, we performed a systematic study of this problem also taking into account the above mentioned theoretical implications of these data. 

Different patterns of defective awareness were predicted as a function of the leading role that ‘absence of language’ and ‘automatic functioning’ could have on the generation of unawareness.

If absence of language is the main determinant of disease unawareness, then patients with the rvSD should show anosognosia for all forms of person-recognition disorders.

If anosognosia is mainly due to the automatic mechanisms underlying the generation of familiarity feelings, then unawareness of face and voice recognition disorders should prevail in patients with the rvSD, whereas anosognosia of name recognition disorders should prevail in those with the lvSD.

If absence of language and presence of automatic mechanisms are equally important for the generation of anosognosia, then patients with the rvSD should be unaware of face and voice recognition disorders, but aware of name recognition defects, due to the opposite in-fluence that ‘absence of language’ and ‘automatic functioning’ have on this form of person-recognition defect.

## 2. Materials and Methods

### 2.1. Participants

A total of 33 patients with right and left temporal variants of Frontotemporal Lobe Degeneration were enrolled at the Cognitive and Behavioural Neurology Unit, Polytechnic University of Marche, Ancona. We will refer to these patients as right (rvSD) and left (lvSD) variants of Semantic Dementia (SD) patients to avoid potential misunderstandings due to the fact that there are well recognized diagnostic criteria for the left variant, now labelled the semantic variant of Primary Progressive Aphasia (svPPA) [45], whereas there are more controversial criteria defining the right variant [46]. According to the classical definition of SD [47], this syndrome is due to a prominent degeneration of the anterior temporal lobes (ATLs) and is characterized by a breakdown of semantic knowledge, with a prevalence of verbal semantic deficits in the lvSD and non-verbal deficits (i.e., prosopoagnosia and picture recognition disorders) in the rvSD [46,47]. 

Clinical diagnoses were made based on clinical history, neuropsychological profile and structural neuroimaging (MRI or CT scan). The patients enrolled fulfilled the international diagnostic criteria for svPPA [45] or showed the clinical features defining the rvSD [46,48].

Inclusion criteria were the following:

- Diagnosis of svPPA or the rvSD, made according to the current criteria;
- Absence of behavioral problems that could interfere with adherence to the experimental battery;
- Availability of a caregiver (familiar or partner or other persons) who lived with the patient or had a daily contact with the patient;
- Availability of a cerebral FDG-PET scan.

Exclusion criteria were the following:

- Absence of a reliable caregiver;
- Absence of a clinical history;
- Objective evidence of hearing loss on audiometry;
- Visual deficits due to ocular disease, uncorrected by spectacles;
- A behavioral problem which could interfere with the administration of a neuropsychological test.

Participant confidentiality precludes public archiving of the data. The data may be accessed upon request to the Scientific Committee of the Neurology Clinic at Marche Polytechnic University (s.luzzi@staff.univpm.it). Interested readers will be required to fill a “Collaboration Statement”.

The SD patients (14 females and 19 males) had a mean age of 72.2 years (SD 8.1) and a mean education of 9.4 years (SD 3.31).

They were classified at the early/moderate stage of the disease, depending on the magnitude of their semantic breakdown according to Julien et al. [49]. The mean illness duration was 3.2 years (SD 1.04; range 1–5 years).

All participants were native Italians who had lived in Italy from birth except a native speaking Englishman who had being living in Italy for 26 years and spoke Italian fluently. All participants gave their written informed consent, and the study was carried out in accordance with the Declaration of Helsinki and was approved by the local Ethics Committee.

### 2.2. Background Neuropsychology

A comprehensive set of domain specific neuropsychological tests was performed in each patient (Table 1). Background neuropsychological data were available for all patients enrolled and included the MMSE [50] as a measure of general cognitive status, the Disyllabic Word Span [51] and the Corsi Blocks [51] to assess short term memory and the delayed recall of the Rey–Osterrieth Complex Figure B [52] to evaluate long term memory. Furthermore, the Luria’s Motor Sequences [52], the Stroop test [52] and the phonological fluency test [51] were used to assess executive functions and attention. The Visual Object and Space Perception Battery [53] was used to evaluate visuo-perceptive and visuo-spatial abilities, whereas constructional praxis was assessed by means of the Rey–Osterrieth Complex Figure B copy version [52] and ideomotor apraxia using the test described by Luzzi et al. [52]. Semantic–lexical functions were assessed using the easy picture naming test, the forced-choice word–picture matching test proposed by Snowden et al. [2] and a test of categorical word fluency [52]. Furthermore, conceptual–semantic associations were studied by means of the verbal and pictorial version of the Pyramids and Palm trees test/PPTT [54].

### 2.3. Experimental Study

The experimental study was aimed at acquiring data relating to the patient’s awareness of their difficulties in recognizing famous people through their faces, voices and names.

**Figure 1:**
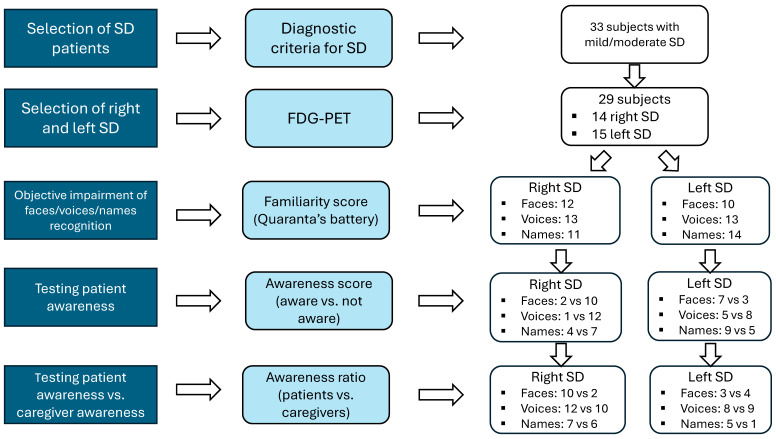
Methodological flowchart.

The methodological flowchart used for patient selection is briefly illustrated in Figure 1. It consists of several steps. The first step seeks to classify subjects into left and right variants of SD based on the distribution of hypometabolism detectable at brain FDG-PET imaging.

The second step intends to identify those patients who objectively show impaired performance in person-specific semantic knowledge for famous people, by means of an experimental battery, previously described and standardized on normal subjects.

In the final step, we investigated self-awareness of the deficit in identifying famous people from their faces/voices/names with an informal questionnaire administered to the patient during the clinical interview and before the neuropsychological assessment was performed. The subjective judgment of person-recognition ability was matched with results obtained on the experimental battery (objective evaluation of anosognosia) and with the corresponding judgment given by the caregiver.

#### 2.3.1. PET Imaging Selection

PET imaging was performed for all patients using the same hardware: a GE Discovery PET/CT 690 VCT scanner, with spatial resolutions of 4.8, 4.8 and 5.0 mm at full-with-half-maximum (FWHM) in the radial, tangential and axial directions, respectively. A three-dimensional emission scan was performed for 15 min, and a post-injection transmission scan was performed using CT for tissue attenuation correction (Helical Full 0.6 s, 3.75 mm, 47 slices).

Two blinded nuclear medicine specialists with expertise in brain imaging were asked to give a referral of the PET imaging. They were asked to indicate the brains’ areas interested by the hypometabolism and if there was a symmetric or asymmetric distribution.

#### 2.3.2. Objective Evaluation of Person Recognition

A battery exploring knowledge of faces, voices and names (the “Famous People Recognition Battery”/FPRB [55] was administered to the patients, using familiarity judgment as a marker of person recognition, for the reasons given in the introduction.

The battery, previously described and standardized the on Italian population [55], is composed of two parts.

The first part is devoted to a preliminary evaluation of the perceptual levels of face and voice processing and is based on two tests of face and voice discrimination:

1. For the discrimination of unknown faces, we used the Benton and Van Allen [56] face recognition test (BFRT), which requires identification of the same or of different unknown faces, seen from different perspectives or under different lighting.
2. For the discrimination of unknown voices we used a new test, formed by twenty stimuli, in which two audio files lasting about 15 s were consecutively presented; the patient was requested to say if the unknown voices belonged to the same or to different persons.

If the patient showed a pathological performance on the perceptual tasks (both tests are standardized on normal subjects) the second part of the battery was not performed.

In the second part of the battery, the patients were required to recognize through their faces, voices and names the same 60 persons. The task consists of recognizing as familiar the face, voice and name of 40 famous persons, distinguishing them from the face, voice and name of 20 unknown persons.

The criterion used to evaluate person specific semantic knowledge was the “familiarity score”, calculated from faces, voices and names of all the 60 famous persons. In this task the patient is shown the face, voice or name of the famous person and is asked to say if that stimulus is familiar or not. The familiarity score was obtained by summing the number of faces, voices or names correctly identified as famous or non-famous (score range 0–60).

In line with the Italian tradition, we transformed raw scores into corrected scores and then to an equivalent score, considering as impaired the performance that, on the familiarity judgment for each modality, corresponded to an equivalent score of 0.

According to this method, patients were classified as showing/non-showing for a person-recognition disorder (defective familiarity judgment) for faces, voices and names.

#### 2.3.3. Self-Awareness of Person-Recognition Disorders

During an informal talk, each patient was asked about their own awareness of any potential deficit related to the recognition of famous persons.

The examiner asked the patient three questions related to knowledge of famous people:

1. Have you ever noticed difficulty recognizing famous people based on their faces?
2. Have you ever noticed difficulty recognizing famous people based on their voice?
3. Have you ever noticed difficulty recognizing famous people based on their name?

Each question was followed by a set of examples in which the examiner tried to let the patient think about real situations involving famous persons. For example, the examiner usually asked if they had difficulty in recognizing the faces of famous persons during TV programs, films, etc.

Similar questions concerned the famous persons’ voices (i.e., “at the radio”), or to the famous person’s name (e.g., “imagine the following situation: you are listening to a television program or the radio, or you are talking about famous people with your family. Has it ever happened to you that you can’t remember the identity of the famous person whose name you’re hearing?”).

The attribution of scores to the three questions asked was made dichotomously. A score of 1 (suggesting some awareness of the deficit) was assigned for each modality (face, voice and name) to positive answers to the corresponding questions, regardless of the strength of the statement. In other words, even if the patient replied that he rarely had difficulty with the recognition of faces, the examiner marked the answer as affirmative and gave a score of 1 (awareness of face recognition). The same procedure was adopted for voices and names.

A score of 0 was assigned if the patient denied any problems in recognizing faces, voices, and names.

We thus obtained three independent scores of awareness for each mentioned variable, i.e., awareness of faces, voices and names.

#### 2.3.4. Caregivers’ Awareness of Person-Recognition Disorders Shown by Their Family Members

During the collection of the clinical history, the caregivers were asked questions, similar to those addressed to the patients and described in the previous section, concerning the patient’s self-awareness of person-recognition disorders through face, voice and name. The attribution of scores was also made dichotomously with 1 (awareness) corresponding to an (at least partially) positive response of the caregiver to the corresponding questions for each modality and the score of 0 given when the caregiver responded that he/she had noticed no difficulties in everyday life in their relatives in recognition of faces, voices or names of known people.

### 2.4. Statistical Analysis

The statistical analyses were carried out with the SPSS statistical package (20.0.0). Since most of the variables were not normally distributed, non-parametric statistics were performed. Mann–Whitney tests was used for continuous variables and chi-squares and the McNemar test were also used on the contingency tables. Cohen’s kappa coefficient was used to measure inter-rater reliability related to the clinical judgement of FDG-PET scans by the two nuclear medicine specialists.

## 3. Results

### 3.1. Demographics and Background Neuropsychology

Demographics and background neuropsychology data of the SD patients are reported in Table 1.

Demographic variables (age, education and gender) were not significantly different in the two groups (right and left SD).

The background neuropsychology data of the lvSD and rvSD showed that patients with a prevalence of left-sided atrophy were significantly more impaired on the verbal version of the PPTT, whereas those showing a right-sided atrophy were significantly more impaired on the pictorial version of the same test. No other significant differences were found between the results of right and left SD patients.

### 3.2. Experimental Study

Results will be reported following the steps of the flow-chart reported in Figure 1.

#### 3.2.1. PET Imaging Selection

On the basis of the clinical judgment of two nuclear medicine specialists, four patients were excluded from the initial group of 33 subjects for the following reasons: for one patient there was no inter-rater agreement and for three patients temporal hypometabolism was scored as symmetrical.

For the remaining 29 SD patients there was agreement about the distribution (areas and asymmetry) of hypometabolism and these patients were considered for further analysis: 14 patients had a prominent right hypometabolism (right SD) and 15 subjects showed a prevalent left hypometabolism (left SD).

Since the selection of patients had been based on the clinical judgment of the two nuclear medicine specialists and not on quantitative data, the k index was calculated to evaluate the inter-rater reliability of this judgment. The k index was 0.94, expressing a high inter-raters concordance and confirming that their judgement was reliable.

#### 3.2.2. Objective Evaluation of Person Recognition from Face, Voice and Name and Exploration of the Corresponding Self-Awareness

Normal or pathological scores obtained on the familiarity judgment score from face, voice and name of the Famous People Recognition Battery [55,56] were used to identify patients who had obtained a performance lower than the cut-off for each of these modalities. Self-awareness was explored with respect to each variable (faces/voice/name) using as reference only patients who had shown an altered familiarity judgement in that modality.

A total of 22 patients (12 right and 10 left SD) presented alterations in the familiarity judgment of faces; 10/12 rvSD and 3/10 lvSD were not aware of their face recognition defect.

Analogously, 26 SD patients (13 rvSD and 13 lvSD) showed an impaired familiarity judgement of voices; 12/13 rvSD and 8/13 lvSD patients were not aware of their voice recognition problem.

Defective familiarity judgement for names was also identified in 25 SD patients (11 rvSD and 14 lvSD); 7/11 rvSD and 5/14 lvSD patients were not aware of their name recognition problem.

**Figure 2:**
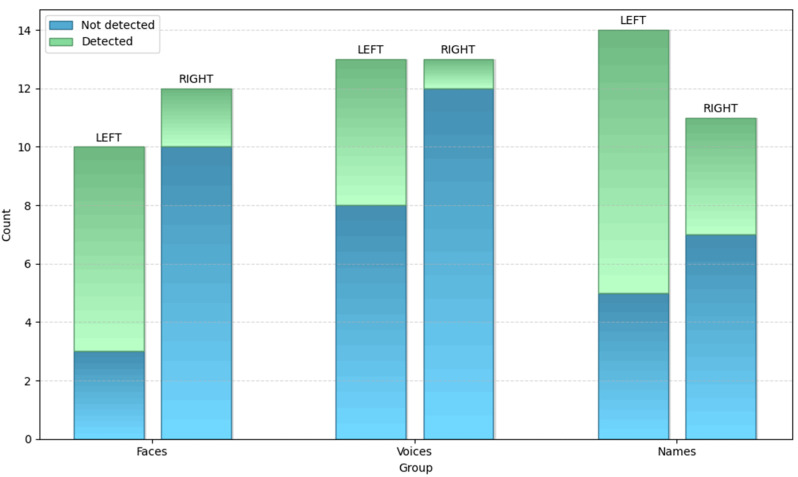
Awareness of defective familiarity judgement for faces, voices and names in right and left SD.

A pictorial representation of data concerning the awareness of defective familiarity judgement for faces, voices and names in patients with left and right variants of SD have been reported in Figure 2.

Statistical analysis gave the following results:

For famous faces, there was a significant difference between subjects with the left or right variants of SD (χ^2^ = 6.42; *p* = 0.01), with right SD patients significantly less aware than left SD patients.

For famous voices the difference between the rvSD and lvSD was not significant (χ^2^ = 3.46; *p* = n.s.), even though the data approached significance (*p* = 0.06), with the rvSD patients being less aware of their problem than the lvSD patients.

For famous names no difference was found between the rvSD and lvSD (χ^2^ = 1.9; *p* = n.s.).

#### 3.2.3. Comparison Between the Caregiver’s and the Patient’s Awareness of Person-Recognition Disorders Through Face, Voice and Name

**Figure 3:**
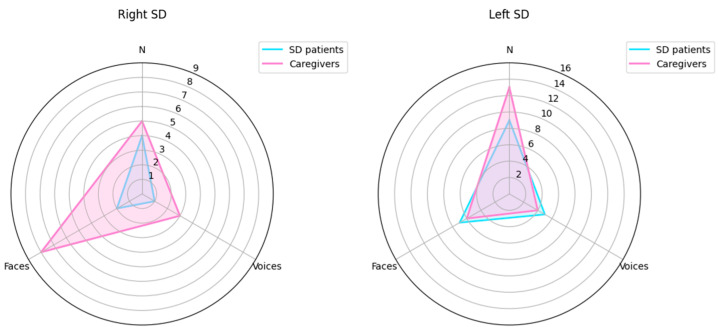
Patient’s awareness and caregivers’ awareness of person-recognition disorders through face, voice and name.

The caregiver’s awareness was compared to the patient’s awareness by means of the McNemar tests in patients with right and left variants of SD. Results are shown in Figure 3.

Within the rvSD group, the following results were found:

Famous faces: 2/12 patients vs. 8/12 caregivers were aware of face recognition disorders shown by the patient. This difference was significant (χ^2^ (1) = 1.2; *p* = 0.03), meaning that the patients were significantly less aware of their problems than the caregivers.

Famous voices: Only 1 patient out 13 and 3 caregivers out 13 were aware of voice recognition disorders shown by the patient. This difference between patients and caregivers was not significant (χ^2^ (1) = 0.32; *p* = 0.62).

Famous names: 4 patients out 11 and 5 caregivers out 11 were aware of name recognition disorders shown by the patient. This difference between patients and caregivers was not significant (χ^2^ (1) = 1.06; *p* = 1.00).

Whitin the lvSD patients, the following results were found:

Famous faces: 7 patients out 10 and 6 caregivers out 10 were aware of face recognition disorders shown by the patient. This difference between patients and caregivers was not significant (χ^2^ (1) = 0.27; *p* = 1.00).

Famous voices: 5 patients out 13 and 4 caregivers out 13 were aware of voice recognition disorders shown by the patient. This difference between patient and caregivers was not significant (χ^2^ (1) = 3.26; *p* = 1.00).

Famous names: 9 patients out 14 and 13 caregivers out 14 were aware of name recognition disorders shown by the patient. This difference between patient and caregivers was not significant (χ^2^ (1) = 0.59; *p* = 0.22); however, patients tended to be less aware than the caregivers of their name recognition defect.

#### 3.2.4. Qualitative Profile of the Caregiver’s and the Patient’s Awareness of Person Recognition Disorders Through Face, Voice and Name

Caregivers’ and patients’ awareness profiles are highlighted in Figure 3.

Caregivers of patients with the rvSD have a greater awareness of the deficit of face recognition shown by their caregiver, whereas caregivers of subjects with the lvSD have a greater awareness of the deficit for names. As regards the awareness of voice recognition disorder, it is interesting to note that the caregivers’ awareness is substantially identical in terms of magnitude to the patients’ awareness in the lvSD, even if caregivers seem to be slightly more aware than the patients with the rvSD. In both cases, the caregivers’ poor awareness of voice recognition disorders might reflect the fact that, in real life, it is difficult for the caregiver to become aware of their relative’s difficulties in person recognition through voice.

The awareness profile of patients with right and left variants of SD is also different, because the right variant shows a lower awareness than the left variant for all modalities of person recognition, even if this prevalence is statistically significant only for faces. 

Looking at the comparison between the caregivers’ and the patients’ profiles, it is interesting to note that only the lvSD patients have a lower awareness than the caregivers for name recognition disorders, even if this difference does not reach statistical significance.

## 4. Discussion

Both clinical and theoretical reasons had motivated the present investigation. From a clinical point of view, the aim was to evaluate the relationship between right-hemisphere damage and unawareness of the disorder. From a theoretical point of view, we aimed to investigate the mechanisms that could promote the right-hemisphere prevalence for dis-ease unawareness. To clarify these issues, we explored unawareness of person-recognition disorders through face, voice and name in patients with the right (rvSD) and left (lvSD) variants of Semantic Dementia.

### 4.1. Unanswered Questions Regarding the Relationships and Underlying Mechanisms That Explain Right-Hemisphere Damage and Lack of Awareness of the Disorder

The relationship between anosognosia and right-hemisphere lesions is still a matter of debate. Some authors have noted the existence of a relationship between anosognosia and right-hemisphere lesions, whereas other authors (e.g., [16,17,18,19,20]) have specified that incidence and severity of anosognosia are particularly high for disorders such as unilateral spatial neglect, that are typically due to right-hemisphere lesions. Results of our investigation could help to clarify the nature of the relation between ‘anosognosia of’ and ‘right hemi-sphere involvement in’ a given task, because face and voice recognition disorders are mainly due to right-hemispheric lesions (e.g., [2,3,4,5,6,7,8,9,10,57,58]), whereas name recognition disorders are mainly due to left-hemispheric lesions [2,3,59,60]. It could therefore be predicted that if a generic link exists between anosognosia and right-hemisphere lesions, unawareness of person-recognition disorders should prevail in patients with the rvSD. If, on the contrary, the right-hemisphere unawareness of a disorder mainly concerns abilities subtended by this hemisphere, then only unawareness of face and voice (but not of name) recognition disorders should prevail in patients with the rvSD.

Regarding the mechanisms that might promote prevalence of the right hemisphere in disease unawareness, a recent general model of hemispheric asymmetries [30,31] states that the main role of the right hemisphere in disease awareness may be linked to two important features of its non-verbal organization (namely its automatic processing and its partly unconscious functioning). The existence of automatic processing modalities in patients with right-hemisphere lesions are documented by the disruption of automatic mechanisms not only in typical right-hemispheric syndrome, i.e., unilateral spatial neglect [30,32,33], but also in prosopagnosia. It is known that face recognition disorders are mainly due to an impaired automatic generation of the face familiarity feelings that trigger the search for corresponding biographic information within the semantic system [4,6,8,10,34]. This greater involvement of the right hemisphere in the automatic aspects of brain functions could influence its level of consciousness. According to Shiffrin and Schneider [35], “auto-matic” mechanisms tend to be associated with less conscious processing whereas “inten-tional/controlled” mechanisms are linked to with more conscious functioning. On the other hand, being that levels of consciousness and intentionality are general aspects of thought that can be shaped by language, the greater role of the right hemisphere in anosognosia could also be due to the absence of language (e.g. [36,37,38,39]). 

These mechanisms are not necessarily mutually exclusive, but can also be complementary. It might be interesting to consider the different predictions that each of these might allow in exploring anosognosia of person-recognition disorders through face, voice and name in the right and left variants of Semantic Dementia, because the automatic generation of familiarity feelings for face and voice recognition is underpinned by the right anterior temporal lobe (rATL), whereas familiarity feelings for name recognition are supported by the left ATL [4,7,10,44].

### 4.2. The Findings of This Study Help Answer Unanswered Questions

The results of our study were evaluated by integrating two orders of data: (a) the com-parison between the subjective awareness and the objective scores obtained by the patient in the corresponding person-recognition modalities and (b) the comparison between the subjective awareness shown by patients and the evaluation provided by their caregivers.

In the comparison between subjective awareness and objective scores obtained by patients in the different person-recognition modalities, the only significant prevalence of anosognosia in patients with the rvSD concerned face recognition disorders. A similar difference approached, but did not reach significance, on voice recognition, whereas no difference was found between the rvSD and lvSD on name recognition. 

In the comparison between the subjective awareness shown by patients and the external evaluation provided by their caregivers, subjects with the rvSD were significantly less aware than caregivers of their difficulties on famous face recognition, but not on their defective recognition of voices or names. On the contrary, patients with the lvSD showed a greater (non-significant) trend to be unaware of their name recognition.

Both these methodologies have thus provided consistent indication that the right hemisphere plays a leading role not only in production [2,3,57,58] but also in the unawareness of face recognition disorders, confirming a critical role of the right hemisphere in this form of anosognosia. On the other hand, less consistent results were obtained for voice and name recognition disorders. In voice recognition, where the right hemisphere also plays a greater role [6,44,61], only the comparison between subjective awareness and objective scores suggested a non-significant prevalence of anosognosia in patients with the rvSD. By contrast, in patients with the lvSD, only the comparison between the subjective awareness shown by the patients and the external evaluation provided by their caregiver suggested a non-significant trend toward a left-hemispheric prevalence of anosognosia for name recognition disorders, which are mainly underpinned by left-hemisphere lesions [44,62,63,64].

### 4.3. Factual and Methodological Reasons Behind the Inconsistencies of Our Results

Several factual and methodological reasons could explain the inconsistencies of these results. An important reason, intrinsic to the nature of person-recognition disorders, is that in daily life person recognition is not independently achieved through face, voice or name, but through the concomitant activation of two or more channels of person identification. Thus, when we meet personally known people (or we see famous people on the TV), we jointly see their faces and hear their voices. Similarly, even when we receive a phone call, we hear a voice, but on the screen we also see the name (or the corresponding phone number) which provides important information about the personal identity of the caller. 

A second reason is that the difficulty of identifying a person through the face, voice and name is different, because several studies have shown that recalling semantic information about known people is more difficult from the voice than from the face [65,66,67,68,69].

A further, methodological reason is that in order to evaluate the unawareness of each of these forms of person-recognition disorders, it was necessary either to match the subjective evaluation of the patient (categorical judgment) with an objective measure of performance (numerical score) or to match the subjective categorical evaluation of the patient with a categorical external evaluation of an informed caregiver. As previously noted, each of the comparisons had some methodological inconsistencies and the solution we selected was to integrate results obtained with these different methodologies. If we take into account all these problems, it is possible to conclude that the consistent prevalence of anosognosia for face recognition disorders proves that anosognosia is particularly frequent for disorders in which the right-hemisphere lesions play a critical role.

Furthermore, since the comparison between the subjective awareness shown by the patients and the external assessment provided by their caregiver leads to a non-significant trend towards a left-hemisphere prevalence of name recognition unawareness, this could support the hypothesis that defective awareness could be at least partly due to the disruption of automatic processing mechanisms. The detection of stimulus familiarity, on which our assessment of the recognition of famous faces, voices and names is based, is in fact an automatic process, and the familiarity feelings of name recognition are supported mainly by the left ATL [7,10,70]. The prevalence of name recognition unawareness in patients with the lvSD could therefore suggest that anosognosia may be at least in part due to a defect in the automatic generation of familiarity feelings and that the hemispheric prevalence of anosognosia may vary according to the side involved in the generation of the corresponding familiarity feelings.

### 4.4. Study Limitations

An important limitation of our study resides in the substantially small sample size of 29 SD patients that, when divided into the two groups of right and left variants, only included 14 rvSD and 15 lvSD patients. This limitation, recognized by the specification “A pilot study” inserted in the title of our work, is due, firstly, to the rarity of this pathology. According to recent data [71], the estimated prevalence of frontotemporal dementia is 10.8/100,000, with SD representing approximately one third of cases [72,73]. Secondly, the need to have PET data to distinguish the rvSD from the lvSD generates a further selection, since this examination is not available for all patients in care.

## 5. Conclusions

This pilot study shows that the prevalence of anosognosia in right-brain-damaged patients is greater for face recognition in which the right hemisphere plays a dominant role. The prevalence of face recognition unawareness in patients with the rvSD suggests that it may be at least in part due to a defect in the automatic processing mechanisms (evocation of familiarity feelings) typical of this hemisphere.

## Figures and Tables

**Table 1:** Demographics and background neuropsychology of the SD patients.

	Right SD	Left SD	Statistics	Significant Differences
*Demographics*				
Age	71.4 (4.9)	72.3 (2.6)	119 *	n.s.
Education	10.7 (4.1)	11.1 (3.6)	120.5 *	n.s.
Sex (M/F)	7/7	9/6	0.29 **	n.s.
*General abilities*				
MMSE (30)	22.7 (4.8)	23 (3.2)	131*	n.s.
RCPM (36)	28.7 (4.3)	27.6 (3.8)	88 *	n.s.
*Memory*				
Bi-syllabic word span	5 (0.7)	4.6 (0.5)	117 *	n.s.
Corsi blocks	4.9 (0.8)	5.1 (0.9)	116 *	n.s.
ROCF-B delayed recall (31)	12.5 (8.4)	14.4 (5.8)	142.5 *	n.s.
*Executive functions*				
LMS (30)	40.4 (9.8)	38.9 (7.8)	61 *	n.s.
Phonological fluency	15.2 (4.8)	17.9 (6.8)	152.5 *	n.s.
Stroop Test time I	41.9 (8.7)	45.1 (10.6)	130 *	n.s.
Stroop Test time II	104.7 (68.7)	127.3 (34.8)	146.2 *	n.s.
Stroop Test errors I	0 (0)	0 (0)	105 *	n.s.
Stroop Test errors II	4.8 (3.1)	3.9 (4.7)	65.5 *	n.s.
*Perceptual-spatial skills*				
VOSP				
Shape detection test (20)	19.7 (0.6)	19.6 (0.4)	116 *	n.s.
Incomplete letters (20)	17.3 (3.5)	16.8 (3.1)	97.5 *	n.s.
Silhouettes (30)	6.2 (3.6)	5.4 (4.1)	93.5 *	n.s.
Object decision (20)	12.4 (3.1)	13.6 (2.9)	114 *	n.s.
Dot counting (10)	9.8 (0.6)	9.8 (0.4)	147 *	n.s.
Number location (10)	7.8 (2.6)	8.3 (1.8)	114 *	n.s.
Position discrimination (20)	17.5 (2.6)	18.1 (1.6)	108 *	n.s.
Cube analysis (10)	9 (1.2)	8.9 (2.1)	117 *	n.s.
*Praxis*				
ROCF-B copy (31)	28.2 (5.4)	26.1 (4.1)	76 *	n.s.
IP (right hand) (20)	20 (0)	20 (0)	105 *	n.s.
IP (left hand) (20)	20 (0)	20 (0)	105 *	n.s.
*Language*				
Verbal fluency	18.6 (7.4)	16.1 (4.8)	52.5 *	n.s.
Naming (40)	24.1 (5.6)	22 (7.9)	99.5 *	n.s.
Reading (40)	39.8 (0.2)	39.4 (0.6)	115 *	n.s.
Single word comprehension (40)	36.5 (5.2)	35.1 (2.3)	80 *	n.s.
PPTT verbal version (30)	22.6 (4.7)	17.3 (1.8)	170 *	*p* < 0.01
PPTT visual version (30)	16.5 (7.5)	23.4 (6.9)	196.5 *	*p* < 0.01

Legend: SD = Semantic Dementia; ns: not significant; MMSE = Mini Mental State Examination; RCPM = Raven’s Coloured Progressive Matrices; ROCF-B = Rey–Osterrieth Complex Figure B; LMS = Luria’s Motor Sequences; VOSP = Visual Object and Space Perception Test; IP = ideomotor praxis; PPTT = Pyramids and Palm Tree Test; * = Mann–Whiteney test; and ** = chi square test.

## Data Availability

The data presented in this study are available on request from the corresponding author due to privacy reasons.

## Abbreviations

The following abbreviations are used in this manuscript:

rATL	right anterior temporal lobe
SD	Semantic Dementia
rvSD	right variant of Semantic Dementia
lvSD	left variant of Semantic Dementia
svPPA	semantic variant of Primary Progressive Aphasia
