# Correction: Luzzi et al. Defective Awareness of Person-Recognition Disorders Through Face, Voice and Name in Right and Left Variants of Semantic Dementia: A Pilot Study. *Brain Sci.* 2025, *15*, 504

**DOI:** 10.3390/brainsci15090912

**Published:** 2025-08-25

**Authors:** Simona Luzzi, Oscar Prata, Guido Gainotti

**Affiliations:** 1 Cognitive and Behavioural Neurology Unit, Department of Experimental and Clinical Medicine, Polytechnic University of Marche, 60126 Ancona, Italy; o.prata@pm.univpm.it; 2 Centre for Neuropsychological Research, Department of Neurosciences, Catholic University of Rome, 00168 Rome, Italy; guido.gainotti@unicatt.it

## 1. Error in Figure 1

In the original publication [1], there was a mistake in Figure 1 in the last two white boxes. The numbers were not corrected because the Figure was revised multiple times during the publication process. The last two white boxes reported the wrong numbers when compared to the text and Figure 3 below.

The two wrong white boxes are as follows:


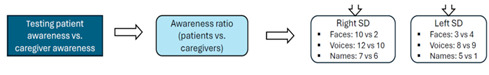


The correct white boxes are presented below, both in isolation and as part of the corrected complete Figure 1.


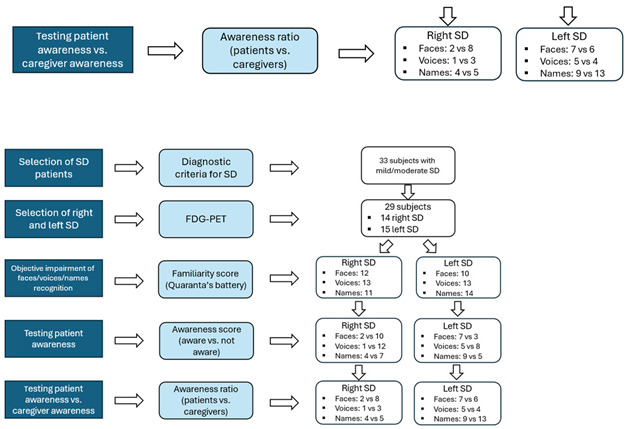


## 2. Error in Figure 3

In the published article, the label “Names” was presented as “N”. This has now been corrected so the label “Names” is presented in the figure, in correspondence with the other two labels “Faces” and “Voices”.

Previous Figure 3 with error:


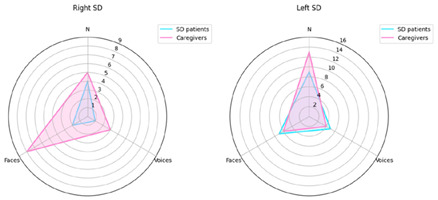


Corrected Figure 3:


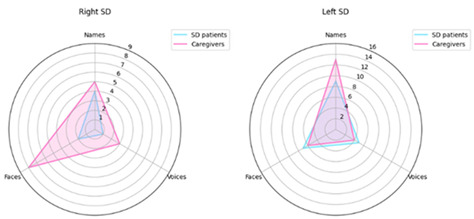


## References

[1] Luzzi S., Prata O., Gainotti G. (2025). Defective Awareness of Person-Recognition Disorders Through Face, Voice and Name in Right and Left Variants of Semantic Dementia: A Pilot Study. Brain Sci..

